# The diagnosis of pulmonary carcinoid using intraoperative fine-needle aspiration cytology: A case report

**DOI:** 10.1016/j.ijscr.2024.110428

**Published:** 2024-10-10

**Authors:** Yuuki Matsui, Koji Takami, Kiyoshi Mori, Yumiko Hirose

**Affiliations:** a Department of General Thoracic Surgery, NHO Osaka National Hospital, Osaka, Japan; b Department of Central Laboratory and Surgical Pathology, NHO Osaka National Hospital, Osaka, Japan

**Keywords:** Pulmonary carcinoid, Intraoperative findings, Fine-needle aspiration cytology

## Abstract

**Introduction and importance:**

Surgeons often need to make intraoperative decisions regarding resection of lung tumors without a preoperative pathological diagnosis. Although intraoperative fine-needle aspiration cytology (FNAC) often provides useful diagnostic information, literatures on its usefulness in pulmonary carcinoids is limited.

**Case presentation:**

A medical chest radiograph revealed an abnormal shadow in the right upper lung field of a 45-year-old Japanese man. Chest computed tomography (CT) revealed a solid 2.5-cm nodule in the right upper lobe. Follow-up CT for one year showed that the tumor size had increased. In case of lung cancer, it was clinically detected to be resectable at stage IA3 with clinical T1cN0M0. Intraoperative FNAC confirmed a specific appearance, and a diagnosis of carcinoid was made. Right upper lobectomy and mediastinal lymph node dissection were performed via video-assisted thoracic surgery. The final histopathological diagnosis was a typical carcinoid with positive chromogranin A, synaptophysin, and CD56, a Ki-67 labeling index of 5 %, and pathological stage IA3 with T1cN0M0, which was consistent with the intraoperative diagnosis.

**Clinical discussion:**

This is the first report describing the diagnosis of pulmonary carcinoid by intraoperative FNAC with the publication of characteristic pathological images, demonstrating the usefulness of intraoperative FNAC.

**Conclusion:**

Intraoperative FNAC may be a low-risk and short-duration procedure for diagnosing pulmonary carcinoids.

## Highlights

• Intraoperative fine-needle aspiration cytology (FNAC) often provides useful diagnostic information for surgical planning.
• There is no literature showing intraoperative pathological images of pulmonary carcinoids using FNAC.
• The cytological appearance of the pulmonary carcinoid detected by intraoperative FNAC in this case was characteristic.
• Intraoperative FNAC may be a low-risk and short-duration procedure for diagnosing pulmonary carcinoids.

## 1 Introduction

Previous reports have shown that nonsmall-cell lung cancer (NSCLC) is often diagnosed during surgical resection, with a preoperative confirmed pathological diagnosis rate of approximately 60 % [1]. The preoperative diagnosis rate of NSCLC at our hospital is approximately 40 %, and pulmonary carcinoids were not preoperatively diagnosed in all previous cases. In such cases, the surgeon must determine the surgical excision method based on an intraoperative pathological diagnosis. We herein report a case in which a typical carcinoid was diagnosed using intraoperative fine-needle aspiration cytology (FNAC) and the information was useful in intraoperative decision-making.

The work has been reported in line with the SCARE criteria and the revised 2023 SCARE guidelines [2].

## 2 Case presentation

**Fig. 1:**
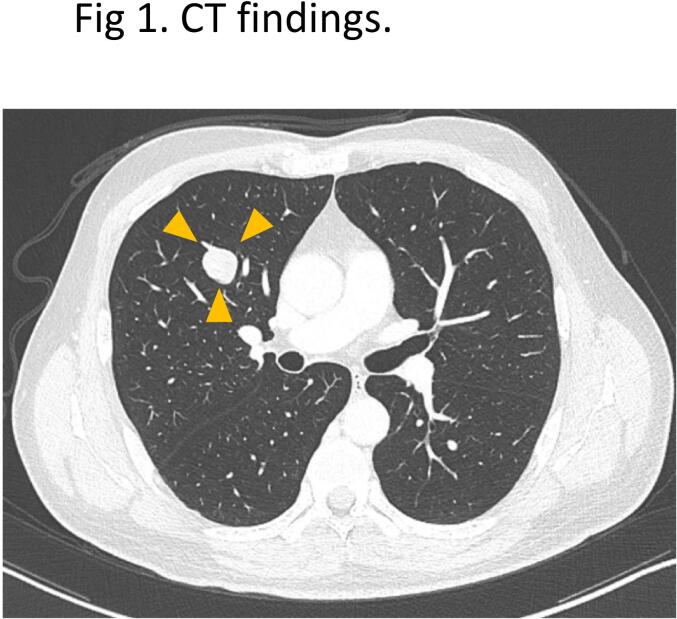
CT findings. CT of the chest shows a well-defined bordering mass of 2.5 × 2.0 cm in S3 of the right lung. The mass extent is indicated by yellow arrow heads. (For interpretation of the references to colour in this figure legend, the reader is referred to the web version of this article.)

A 45-year-old Japanese man presented with an abnormal shadow in the right upper lung field on a chest radiograph obtained during a medical examination. The patient was completely asymptomatic and his medical history was unremarkable. Chest computed tomography (CT) revealed a mass in the S3 lesion of the right lung (Fig. 1). Given its maximum diameter of 2.5 cm and solid diameter of 2.1 cm, the mass was suspected to be a hamartoma and was followed up with CT for 1- year. However, the mass grew and could not be ruled out as a malignant tumor; therefore, a pathological diagnosis was necessary.

**Fig. 2:**
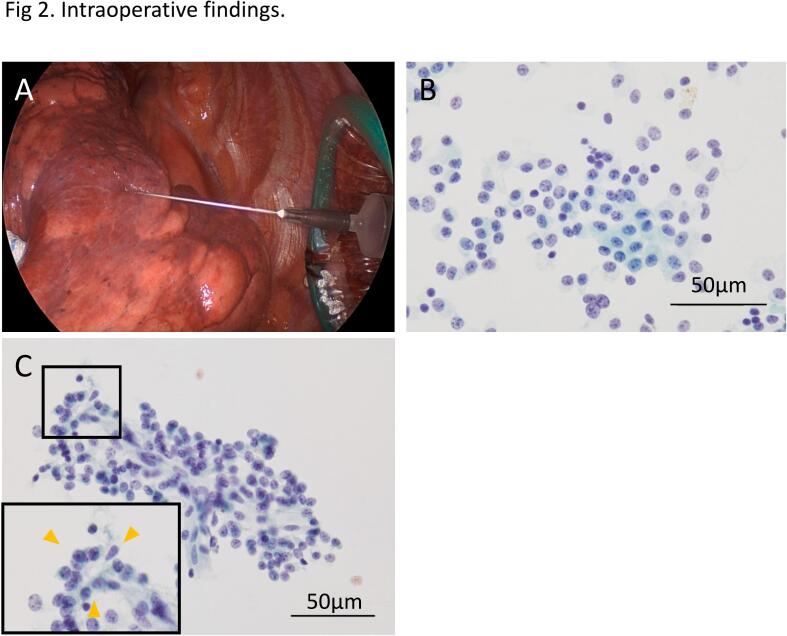
Intraoperative findings. (A) Image of fine-needle aspiration cytology in the chest, (B) Fine needle aspiration cytology. Round nuclear and coarse granule salt-and-pepper chromatin patterns. Papanicolaou staining. (C) Fine-needle aspiration cytology. Rosette-like formations are indicated by yellow arrow heads. Papanicolaou staining. (For interpretation of the references to colour in this figure legend, the reader is referred to the web version of this article.)

**Fig. 3:**
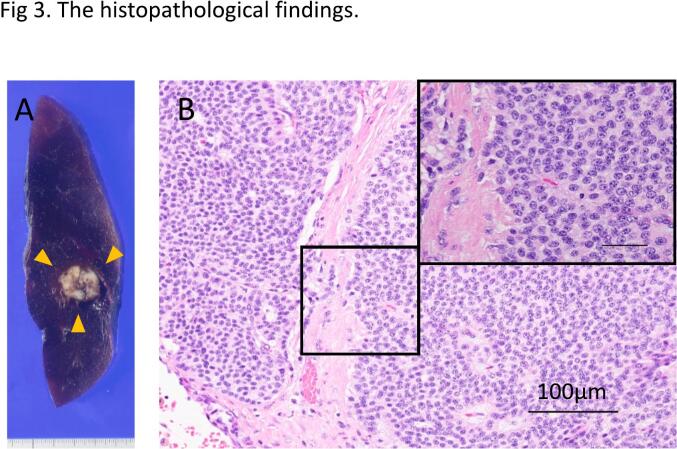
The histopathological findings. (A) Macroscopic findings of the formalin-fixed resection specimen. Macroscopic findings showed the mass with a maximum diameter of 2.1 × 1.7 cm and invasive diameter of 2.1 cm in the right upper lobe. The tumor extent is indicated by yellow arrow heads. (B) The histopathological examination revealed that the tumor was composed of a proliferation of atypical cells with round nuclear and stippled chromatin without mitosis or necrosis. (For interpretation of the references to colour in this figure legend, the reader is referred to the web version of this article.)

**Fig. 4:**
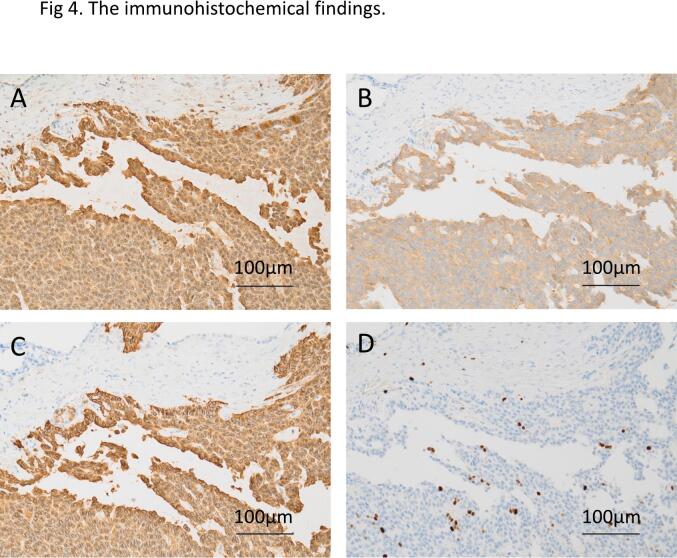
The immunohistochemical findings. Immunohistochemical staining demonstrated positivity for (A) Chromogranin A, (B) Synaptophysin, and (C) CD56 and (D) Ki-67 labeling index is about 5 %.

Preoperative transbronchial lung biopsy (TBLB) was not performed because of the lack of a selective bronchus for the tumor on CT. 18-F-fluorodeoxyglucose (FDG) positron emission tomography (PET)/CT was not performed because of patient refusal. If the mass was indeed a lung cancer, it was clinically determined to be resectable at stage IA3 with clinical T1cN0M0. Because the tumor was deep-seated, we planned to perform surgical resection after making a definitive diagnosis using intraoperative FNAC. Intraoperative FNAC was performed as follows: First, the tumor was palpated on the lung surface to confirm its location. Next, a 22-gauge needle was inserted into the specimen, a syringe attached to the needle was then rapidly pulled to create negative pressure at the puncture site, and thereafter a small amount of tissue was extracted through the needle. The needle was then removed from the syringe, the syringe tube was pulled to allow air to enter, the needle was reattached, and the smear specimen was blown onto a glass slide. This process was repeated twice. To improve diagnostic accuracy, the inside of the needle was rinsed with saline, the cells were centrifuged from the washing solution, and a slide was prepared. FNAC suggested that it was a neuroendocrine tumor consisting of many cells with round nuclear and coarse granular salt-and-pepper chromatin patterns and some cells with rosette-like formations (Fig. 2). Based on the above findings, the patient was diagnosed with carcinoid, and right upper lobectomy and mediastinal lymph node dissection were performed using video-assisted thoracoscopic surgery. A formalin-fixed resected specimen showed a smooth margined mass with a maximum diameter of 2.1 × 1.7 cm in the right upper lobe. A histopathological examination showed that the tumor was composed of proliferating atypical cells with round nuclei and stippled chromatin. There was no pleural, lymphatic, or vascular invasion of the tumor cells and no mitosis or necrosis (Fig. 3). Immunohistochemical staining revealed positivity for chromogranin A, synaptophysin, and CD56, with a Ki-67 labeling index of 5 % (Fig. 4). The final histopathological diagnosis was a typical carcinoid with a maximum diameter of 2.1 × 1.7 cm and invasive diameter of 2.1 cm, at pathological stage IA3 with T1cN0M0, which was consistent with the intraoperative diagnosis. The patient was followed without adjuvant chemotherapy and survived without recurrence for 24 months.

## 3 Discussion

There have been few reports of cases diagnosed with pulmonary carcinoid by intraoperative FNAC [3]. This is the first report to describe the diagnosis of pulmonary carcinoid using intraoperative FNAC with characteristic pathological images.

Regarding the diagnosis of lung tumors, methods to obtain a preoperative pathological diagnosis include a TBLB and CT-guided percutaneous lung biopsy (CTGLB). A TBLB requires the presence of a selective bronchus to the tumor, whereas a CTGLB is indicated when the tumor is not deep-seated. Detterbeck et al. reported that a TBLB can be used to diagnose carcinoids in 70 % to 80 % of patients with an adequate specimen [4]. On the other hand, there are methods of obtaining an intraoperative diagnosis, including FNAC and wedge resection, and intraoperative FNAC is less costly than wedge resection because wedge resection requires stapling instruments. In addition, the time required to process the specimens is shorter for cytology than for histology. Furthermore, wedge resection is often difficult when the target is deep-seated. Regarding the palpation of lung tumors via video-assisted thoracic surgery with minimum thoracotomy, the tumor can be palpated by the assistant surgeon moving the lung closer to the operator's side, except when the lung tumor is located very close to the pulmonary hilum. In the present case, there was no selective bronchus for the tumor, and the tumor was deep-seated; therefore a CTGLB and wedge resection were not unlikely indicated, and intraoperative FNAC was deemed appropriate.

Carcinoids exhibit typical morphologic features, such as palisading arrangements, trabecular formations, or rosette-like formation and are composed of uniform tumor cells with eosinophilic cytoplasm, inconspicuous nucleoli, and fine granular chromatin [5]. There are some reports in which the characteristic morphology of a carcinoid is detected by cytology in a TBLB [6], and a carcinoid diagnosis is made. In the present study, we diagnosed carcinoids by intraoperative FNAC, capturing features similar to the characteristic images of cytology in a TBLB. It has been previously reported that carcinoids were diagnosed in 20 %–76.5 % of cases by intraoperative FNAC [3,7,8], but intraoperative pathological images were not published among them. This report includes the findings of intraoperative pathological images, which may positively contribute to the intraoperative diagnosis of carcinoids.

Although a CTGLB is considered among the preoperative diagnostic procedures, it carries some risks, such as pneumothorax, air embolization, and pleural seeding [9,10]. Hata et al. reported 1 case (1 %) of grade 3 pneumothorax and 1 case (1 %) of grade 4 air embolization as serious complications among 111 patients who underwent a CTGLB for a preoperative examination of NSCLC [10]. In contrast, intraoperative FNAC associated with a low-risk of pulmonary hemorrhage, and seeding of tumor cells [11].

Finally, several limitations associated with intraoperative FNAC should be mentioned. First, the diagnostic accuracy of intraoperative FNAC for lung tumors has been reported to be 88–92 % [12,13], which is considered to be a relatively high success rate. However, it is necessary to note that the correct diagnosis may be missed in approximately 10 % of the cases. Second, intraoperative FNAC is a good indication if the tumor is deep-seated and located far from the pulmonary hilum. However, if it is very close to the hilum, then it is difficult to palpate the tumor and safely puncture it in order to avoid damaging any blood vessels.

In summary, pulmonary carcinoids can be diagnosed by intraoperative FNAC, suggesting that intraoperative diagnostic procedures such as FNAC could be used for making a diagnosis without much preoperative effort and with a lower risk than with a CTGLB.

## 4 Conclusion

This is the first report to describe the diagnosis of pulmonary carcinoids using intraoperative FNAC based on the findings of characteristic pathological images. According to our findings, we conclude that intraoperative FNAC is a low-risk and short-duration procedure for determining the diagnosis of lung tumors and that it may be worthwhile to try this method first, since it has been suggested that it may also be useful in the diagnosis of pulmonary carcinoid.

## Author contribution

Yuuki Matsui wrote the original draft, reviewed, and edited the manuscript. Koji Takami supervised, wrote, reviewed, and edited the manuscript. Kiyoshi Mori and Yumiko Hirose: Pathological diagnosis. All authors have read and approved the final manuscript.

## Consent

Written informed consent was obtained from the patient for publication of this case report and accompanying images. A copy of the written consent is available for review by the Editor-in-Chief of this journal on request.

## Ethical approval

Not applicable (data and figure included does not compromise the identity of the patient).

## Guarantor

Corresponding author: Koji Takami.

## Research registration number

None.

## Funding

The authors declare that they have no disclosure of financial interests.

## Conflict of interest statement

The authors declare that they have no competing interests.

## Data Availability

The authors declare that the data in this article are available within the article.
